# Switch from planktonic to sessile life: a major event in pneumococcal pathogenesis

**DOI:** 10.1111/j.1365-2958.2006.05310.x

**Published:** 2006-08-01

**Authors:** Marco R Oggioni, Claudia Trappetti, Aras Kadioglu, Marco Cassone, Francesco Iannelli, Susanna Ricci, Peter W Andrew, Gianni Pozzi

**Affiliations:** 1 Laboratorio di Microbiologia Molecolare e Biotecnologia, Dipartimento di Biologia Molecolare, Università di Siena Siena, Italy; 2 UOC Batteriologia, Azienda Ospedaliera Universitaria Senese Siena, Italy; 3 Department of Infection, Immunity and Inflammation, University of Leicester Leicester, UK

## Abstract

Two main patterns of gene expression of *Streptococcus pneumoniae* were observed during infection in the host by quantitative real time RT-PCR; one was characteristic of bacteria in blood and one of bacteria in tissue, such as brain and lung. Gene expression in blood was characterized by increased expression of pneumolysin, *pspA* and *hrcA*, while pneumococci in tissue infection showed increased expression of neuraminidases, metalloproteinases, oxidative stress and competence genes. *In vitro* situations with similar expression patterns were detected in liquid culture and in a newly developed pneumococcal model of biofilm respectively. The biofilm model was dependent on addition of synthetic competence stimulating peptide (CSP) and no biofilm was formed by CSP receptor mutants. As one of the differentially expressed gene sets *in vivo* were the competence genes, we exploited competence-specific tools to intervene on pneumococcal virulence during infection. Induction of the competence system by the quorum-sensing peptide, CSP, not only induced biofilm formation *in vitro*, but also increased virulence in pneumonia *in vivo*. In contrast, a mutant for the ComD receptor, which did not form biofilm, also showed reduced virulence in pneumonia. These results were opposite to those found in a bacteraemic sepsis model of infection, where the competence system was downregulated. When pneumococci in the different physiological states were used directly for challenge, sessile cells grown in a biofilm were more effective in inducing meningitis and pneumonia, while planktonic cells from liquid culture were more effective in inducing sepsis. Our data enable us, using *in vivo* gene expression and *in vivo* modulation of virulence, to postulate the distinction – from the pneumococcal point of view – between two main types of disease. During bacteraemic sepsis pneumococci resemble planktonic growth, while during tissue infection, such as pneumonia or meningitis, pneumococci are in a biofilm-like state.

## Introduction

*Streptococcus pneumoniae* is the main cause of community-acquired pneumonia and meningitis in children and the elderly and of septicaemia in HIV-infected individuals. It is also one of the principal causes of otitis media. Bacteraemia has been reported to accompany more than 25% of cases of pneumococcal pneumonia (Afessa *et al*., 1995) and pneumococcal bacteraemia accounts for at least 10% of all cases of bacteraemia (Aszkenasy *et al*., 1995). In places with high HIV positivity, there is a significant increase in the rate of pneumococcal bacteraemia and the increase is most marked in young adults (Karstaedt *et al*., 2001). In addition to these more classical situations, pneumococci are also reported in a variety of other diseases, including arthritis, osteomyelitis, endocarditis, endophthalmitis, abscesses, necrotizing fasciitis, and sinusitis. Despite its importance as a pathogen, relatively little is known about the pathophysiology of pneumococcal diseases. Unsolved issues include (i) the mechanism of shifting from a colonizer organism to an invader, (ii) the mechanism of translocation across the blood–brain barrier to cause meningitis, (iii) the difference between sepsis with a primary focus of disease and sepsis without focal disease and (iv) differences in bacterial behaviour during the diverse clinical diseases.

Soon after the discovery of *S. pneumoniae* and the setting up of a serological classification scheme, it became obvious that pneumococcal strains tended toloose characteristic phenotypes upon cultivation in laboratory media. The link between colony phenotype and virulence was first described in the seminal study of Griffith in 1928 (Griffith, 1928). He introduced a completely new concept, namely transformation of type. These experiments led to the mouse not only being used for selection of revertants, but more importantly to enable competence for genetic transformation and selection of transformants. Only a few further reports on transformation *in vivo* were published, such as the work of Austrian, who described induction of competence in a variety of mammals, including primates (Austrian, 1952) and that of Conant and Sawyer who demonstrated that competence can occur in distinct anatomical sites, including the lung and peritoneum (Conant and Sawyer, 1967).

Some 30 years after the description of the methodology for *in vitro* transformation (Alloway, 1932), Tomasz and Hotchkiss (1964) reported that co-ordinated induction of competence takes place at a particular cell density in exponentially growing cultures of pneumococci. Later, Tomasz showed that control of the competent state in the pneumococcus is dependent on a hormone-like pneumococcal product (Tomasz, 1965). The identification of a unmodified 17-amino-acid peptide pheromone (competence stimulating peptide; CSP) as responsible for the cell-to-cell signalling in competence development enabled a detailed description of this quorum-sensing phenomenon (Havarstein *et al*., 1995). A recent analysis using microarrays described the temporal expression profiles of pneumococcal genes upon exposure to CSP (Dagkessamanskaia *et al*., 2004; Peterson *et al*., 2004). Peterson and colleagues described early, late and delayed gene induction and repression after CSP stimulation. Genes showing early expression include the *comABCDE* genes and the alternative sigma factor *comX*. The late *comX*-dependent genes included further genes playing important roles in transformation, while part of the delayed *comX*-independent genes appear to be stress related. The group of repressed genes included ribosomal protein loci and other genes involved in protein synthesis. In these studies no change in the expression of the most obvious virulence genes was found (Dagkessamanskaia *et al*., 2004; Peterson *et al*., 2004).

A variety of virulence factors have been described for *S. pneumoniae,* including the polysaccharide capsule (Bornstein *et al*., 1968), the pore-forming toxin pneumolysin and a variety of surface proteins including PspA, PspC, neuraminidases and hyaluronidase and the zinc metalloproteinases (Kadioglu *et al*., 2002; Ogunniyi *et al*., 2002; Blue *et al*., 2003; Chiavolini *et al*., 2003; Iannelli *et al*., 2004; King *et al*., 2004; Manco *et al*., 2006). For most of these factors, the impact on virulence differed when mutants were assayed in different models of infection. The tissue or disease restricted importance of single bacterial factors highlights the fact that *in vivo* situations have distinct constraints that impact on fitness. This concept is also supported by genome-wide screenings for virulence factors and microarray analysis for virulence genes, which demonstrate the tissue specific gene expression of a wide variety of genes (Polissi *et al*., 1998; Lau *et al*., 2001; Hava and Camilli, 2002; Orihuela *et al*., 2004a,b; LeMessuier *et al*., 2006).

While pneumococcal virulence and transformation have been studied for a long time, the physiological states of the pneumococcus *in vitro* have been studied less intensely. One reason for this is that pneumococci tend to undergo autolysis after prolonged incubation. Therefore, there is a lot of information on autolysis but scarce information on how the pneumococcus behaves in different *in vitro* environments, such as planktonic growth or sessile growth on agar or in submerged biofilms. However, some characteristics of biofilm formation by oral streptococci, which are taxonomically very similar to the pneumococcus, have been described. Interestingly, the formation of biofilm is, at least to a certain extent, linked to competence in *S. gordonii*, *S. mutans* and *S. intermedius* (Loo *et al*., 2000; Li *et al*., 2002; Cvitkovitch *et al*., 2003; Petersen *et al*., 2004; Qi *et al*., 2006). In these bacteria, biofilm is induced by the CSP peptide, and competence mutants in *comC* (CSP peptide gene), *comD* (CSP receptor histidine kinase) or *comE* (regulator of the to ComD kinase) are biofilm defective (Loo *et al*., 2000; Li *et al*., 2002; Cvitkovitch *et al*., 2003; Petersen *et al*., 2004; Qi *et al*., 2006). For *S. pneumoniae*, few data were available on biofilm formation until recently, when a biofilm reactor-grown biofilm was reported, and the extracellular polymeric substance of the pneumococcal biofilm was described (Waite *et al*., 2001; Donlan *et al*., 2004; Allergucci *et al*., 2006).

We have recently published that CSP, if given intravenously in a murine model of pneumococcal bacteraemia, increases the survival rate of mice (Oggioni *et al*., 2004). This antibacterial or anti-fitness effect was in part explained by a bacteriostatic effect that CSP has on logarithmically growing cultures (Dagkessamanskaia *et al*., 2004; Oggioni *et al*., 2004; Qi *et al*., 2006). As it was difficult to combine these observations to the historical data on transformation *in vivo,* we more systematically explored the behaviour of the competence network *in vitro* and *in vivo.* In the present work, by analysing gene expression profiles and exploiting models of infection, we were able to identify two different physiological conditions of pneumococci during infection of the host and to correlate them to the *in vitro* models of liquid growth and biofilm.

## Results

### Real time RT-PCR analysis of pneumococcal gene expression profiling during infection

With the aim to define gene expression pattern that may characterize pneumococcal physiology during infection, we set up a method of quantitative gene expression analysis in mice. In order to permit evaluation of samples from individual mice, without the need for pools, we used real time RT-PCR (Oggioni *et al*., 2004). A set of 29 genes to be monitored was decided after reviewing the literature for known virulence-related genes, competence genes and other regulators (Polissi *et al*., 1998; Echenique and Trombe, 2001; Lau *et al*., 2001; Kadioglu *et al*., 2002; Hava *et al*., 2003; Mascher *et al*., 2003; Oggioni *et al*., 2003; 2004; Dagkessamanskaia *et al*., 2004; King *et al*., 2004; Peterson *et al*., 2004) (Table 1). Virulence and virulence-related genes were those coding for the capsule, the surface proteins PspA and PspC, the neuraminidases NanA and NanB, the surface proteinases IgA, ZmpB, ZmpC and PrtA, the autolysin, the pore-forming toxin pneumolysin and genes related to oxidative stress, such as *sodA* and *nox* and cell wall biosynthesis *ddlA* and *pbp2x*. Expression of the competence system was assayed with primers for *comA* (ABC transporter for CSP export and maturation), *comE* (regulator of CSP specific two-component system) and *comX* (alternative sigma factor). Other regulators assayed were the two-component systems TCS02 (*micAB*), TCS04 (*pnpSR*), TCS05 (*ciaRH*) and TCS13 (*blpRH* or *spiRH*), the putative quorum-sensing gene *luxS*, the heat shock regulators *hrcA* and *ctsR*, the serine/threonine protein kinase *stkP*, the virulence gene regulator *mgrA*, the putative iron-regulated regulator SP1638 (*dtxR* or *smrB*) and the putative sugar regulators SP1899 (*msmR* or *araC*) and *regR* (Table 1).

**Table 1:** Relative quantification of pneumococcal gene expression.

		Fold change of pneumococcal gene expression^a^				
		
Gene		Brain	Lung	Blood	Agar	Biofilm
Virulence genes						
*ply*	SP1923	0.3(0.2)	0.2(0.1)	1.3(0.3)	0.01(0.01)	0.2(0.1)
*pspA*	SP0117	0.3(0.1)	0.3(0.1)	1.3(0.2)	0.4(0.1)	0.2(0.1)
*cps4A*	SP0346	2.2(0.9)	1.6(0.8)	1.2(0.2)	7.0(1.8)	1.6(0.4)
*pspC*	SP2190	1.5(0.9)	1.2(0.2)	1.8(0.4)	0.7(0.1)	0.6(0.4)
*nanA*	SP1693	18.8(12.0)	16.6(10.2)	1.3(0.7)	0.3(0.1)	59.4(28.0)
*nanB*	SP1687	3.8(0.9)	3.2(0.7)	2.0(0.3)	0.3(0.2)	4.8(1.1)
*igA*	SP1154	3.3(0.8)	2.5(0.7)	1.1(0.5)	0.3(0.1)	nd
*zmpB*	SP0664	2.2(0.9)	2.2(1.6)	1.2(0.2)	0.3(0.1)	nd
*zmpC*	SP0071	5.2(1.6)	4.1(0.9)	1.4(0.3)	0.3(0.1)	nd
*sodA*	SP0766	10.4(3.1)	13.9(10.8)	1.5(0.6)	1.7(0.9)	nd
*nox*	SP1469	7.4(4.8)	8.7(9.2)	1.0(0.3)	1.3(0.8)	0.07(0.02)
*lytA*	SP1937	2.0(1.1)	1.3(0.2)	2.3(0.4)	2.5(0.2)	nd
*prtA*	SP0641	0.8(0.5)	0.8(0.2)	1.3(0.2)	0.2(0.1)	0.4(0.3)
*ddlA*	SP1671	nd	1.8(0.3)	2.4(0.8)	nd	4.0(0.6)
*pbp2x*	SP0336	nd	6.3(2.2)	1.3(0.4)	nd	7.7(2.3)
Regulators						
*comA*	SP0042	10.0(1.5)	7.1(1.0)	1.3(0.4)	2.0(1.2)	7.1(0.7)
*comE*, *tcs12*	SP2235	6.0(2.1)	4.1(1.5)	1.3(0.3)	1.3(0.5)	nd
*comX*	SP0014	12.0(8.4)	7.3(4.2)	1.6(0.3)	1.1(0.1)	5.1(0.8)
*micA*, *tcs02*	SP1227	2.2(1.2)	1.4(0.6)	2.2(0.5)	1.2(0.6)	1.8(0.6)
*pnpR*, *tcs04*	SP2082	3.3(1.0)	3.1(0.7)	2.0(0.4)	1.7(0.9)	4.4(1.3)
*ciaR*, *tcs05*	SP0798	1.7(0.8)	1.3(0.4)	1.5(0.3)	1.9(0.7)	3.6(0.3)
*blpR*, *tcs13*	SP0526	5.4(1.0)	4.9(2.2)	3.5(0.6)	0.4(0.2)	4.0(1.9)
*luxS*	SP0340	5.0(1.4)	4.3(0.6)	2.4(0.4)	5.01(0.4)	2.8(0.3)
*hrcA*	SP0515	0.1(0.1)	0.1(0.1)	1.2(0.1)	0.2(0.1)	0.1(0.03)
*ctsR*	SP2195	nd	4.8(2.8)	1.6(0.5)	2.8(1.6)	nd
*mgrA*	SP1800	31.7(12.8)	18.7(9.8)	4.9(0.8)	47.4(21.9)	15.8(7.7)
*regR*	SP0330	4.0(2.8)	5.1(2.5)	3.4(0.7)	8.5(8.0)	3.1(0.8)
*stkP*	SP1732	3.9(2.1)	5.9(6.9)	5.3(3.7)	0.4(0.4)	1.4(0.1)
*dprA*	SP1266	12.2(5.9)	7.7(6.9)	0.9(0.7)	0.06(0.03)	nd
*dtxR*	SP1638	10.1(5.9)	11.7(9.1)	1.0(0.4)	0.5(0.3)	nd
*msmR*	SP1899	8.2(5.6)	5.6(3.1)	0.6(0.29)	0.03(0.04)	2.23(0.6)

a. Fold change in gene expression measured by quantitative real time RT-PCR was calculate using the 2^–^^ΔΔ^^CT^ method (Livak and Schmittgen, 2001). The internal control gene was gyrB and the reference condition was liquid culture in mid exponential phase. The mean and standard deviation was calculated on the data from three to five infected mice assayed individually. nd, not done.

**Fig. 1:**
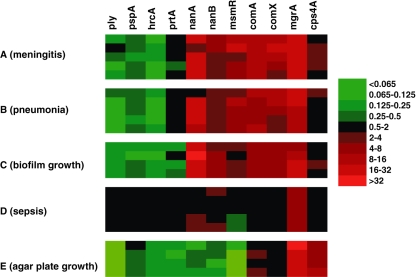
*In vivo* and *in vitro* gene expression patterns of selected *S. pneumoniae* genes. mRNA levels were measured by quantitative real time RT-PCR and evaluated according to the 2^–ΔΔCT^ method (Livak and Schmittgen, 2001). Gene expression of strain TIGR4 was analysed in six distinct conditions which included (A) pneumococci recovered from brain homogenates of five mice infected IC, (B) pneumococci recovered from lung washes of five mice infected IN, (C) four pools of bacteria forming biofilm on plastic, (D) pneumococci recovered from blood samples of five IV infected mice, and (E) pneumococci on agar plates. All values of fold change in gene expression are reported as change towards expression of the relative gene in liquid culture (black colour indicates similar expression as in liquid). Each line in the figure represents a different biological replica, while each column represents a single gene. The colour code used is displayed on the right.

The gene expression of strain TIGR4 during infection was monitored by real time RT-PCR in three distinct *in vivo* models, namely meningitis after intracranial (IC) challenge, pneumonia after intranasal (IN) challenge and sepsis after intravenous (IV) challenge. Brain samples from mice with meningitis, lung washes of mice with pneumonia and blood of mice with sepsis were collected at the onset of severe symptoms and analysed individually. Pneumococcal gene expression during infection in mice showed two principal patterns; one found in the blood and one during infection of lungs and brain (Fig. 1 and Table 1). The overall pattern of gene expression in the blood of mice was very similar to the pattern of gene expression in liquid tryptic soy broth (TSB) medium during exponential phase of growth *in vitro*. Values of fold change of gene expression in blood are between 0.5 and 2 (non-significant variation) and indicate a reduced variation in this condition, with respect to the reference condition of growth liquid medium. While different from blood, the relative gene expression values of pneumococci in tissue of infected mice, either lung or brain after direct inoculation, are similar. When comparing gene expression in blood against gene expression of pneumococci isolated from lungs and brain it is obvious that some genes show marked variations. A striking feature is that in the tissues each of the three competence genes (*comA*, *comE*, *comX*) had much elevated expression compared with sepsis. The expression of other regulators and virulence genes varied according to site. The virulence genes *igA*, *nanA*, *nanB*, *nox*, *sodA*, *pbp2x*, *zmpB* and *zmpC* were upregulated in tissues, whereas *ply* and *pspA* showed increased expression in blood. Also regulator genes were differentially expressed, for example *comE*, *comX*, *ctsR*, *dtxR*, *msmR* and *regR* were more expressed in tissues, while the major heat shock regulator gene, *hrcA*, was upregulated in blood. Some of the monitored genes, namely *blpR*, *ddlA*, *luxS*, *mgrA*, *pnpR* and *stkP* showed increased expression in all *in vivo* conditions over liquid culture in TSB (Fig. 1 and Table 1).

In all three models of infection samples were collected from animals exhibiting severe signs of disease. In order to evaluate gene expression patterns of pneumococci earlier during infection, samples were taken at 6, 12 and 24 h after IN challenge. Relative gene expression patterns determined were indistinguishable from those detected in lung samples taken at the onset of severe symptoms (data not shown).

In addition to the *in vitro* reference condition of exponentially growing bacteria in liquid medium, we also assayed for gene expression of pneumococci on blood agar plates. On agar, the *cps4A* gene, unchanged in all other conditions, showed a significant upregulation, while most other genes showed no changes or diminished levels of expression (Table 1). In summary, the results indicate that gene expression patterns of pneumococci in the blood of septic mice are indistinguishable from those in *in vitro* liquid exponential culture and that a completely different gene expression pattern was observed when analysing pneumococci from infections of brain or lung. None of the two *in vivo* gene expression patterns matched that found in the *in vitro* condition of growth on agar plates and none of the two *in vitro* conditions matched that found in tissues.

### Development of a pneumococcal biofilm model

**Fig. 2:**
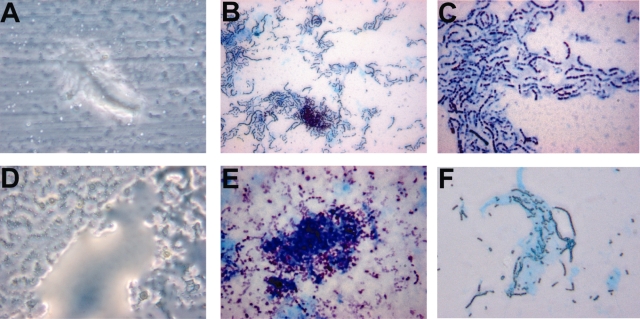
Light microscopy of pneumococcal biofilm. Appearance of pneumococci attached to plastic microtiter plates in the presence of CSP. Panels A, B and C refer to strain D39, while panels D, E and F to TIGR4. Panels A and D show the glossy amorphous blebs visible by phase contrast microscopy on the bottom of microtiter wells. The images of the structures sticking to the bottom of the microtiter wells were taken from washed, but non-fixed, 6 well plates with a 40x objective. Panels B and E show methanol fixed samples stained with alcian blue for polysaccharides and counterstained with crystal violet and observed in bright filed (40× objective). Panels C and F are as B and E, but observed with a 100× objective. Noteworthy is the uneven distribution of pneumococci on the bottom of the wells (B, C, E and F), which is compatible with the structures seen in panels A and D. The blue staining surrounding pneumococci in bright field (panels B, C, E and F) is compatible with a polysaccharide extracellular matrix described generally as main constituent of bacterial biofilms.

In the search for a novel *in vitro* condition, possibly matching the gene expression pattern in tissues, a microtiter model for the assay of pneumococcal biofilm formation was set up. Attachment to plastic of pneumococcal strains TIGR4, carrying a *comC*2 allele coding for CSP2, and D39 (*comC*1, CSP1) was evaluated in flat bottomed polystyrene wells after 18 h of incubation. Direct microscopic examination of the bottoms of the wells after repeated washes showed the presence of pneumococci attached to the plastic support when incubated in CSP-supplemented medium (CSP1 30 ng ml^−1^ for D39; CSP2 100 ng ml^−1^ for TIGR4). Samples were observed both as wet preparations by phase contrast microscopy and after fixation with methanol and staining in bright field. Bacterial cells covered the whole bottom of the wells and were unevenly distributed forming various types of aggregates and clusters (Fig. 2). Staining by alcian blue, a dye used for polysaccharide matrixes, revealed extracellular material around the attached bacterial cells (Fig. 2B, C, E and F). The microscopic appearance of both strains was similar, and in the case of D39, attached cells tended, in some experiments, to form short chains. Phase contrast microscopy revealed amorphous ‘bleb-like’ structures compatible with the appearance of hydrated biofilms (Fig. 2A and D). The unencapsulated derivatives RX1 and FP23 of the above strains formed comparable structures as the encapsulated wild-type strains (data not shown). Neither attached cells nor biofilm structures were visible in wells incubated without CSP.

Gene expression of TIGR4 pneumococci attached to plastic was assayed after 18 h of incubation. All genes assayed showed the same pattern of expression in attached cells as in cells recovered from animals with pneumonia or meningitis (Fig. 1C). This similarity in gene expression pattern was mostly evident for those genes that showed the greatest variation with respect to the reference values (liquid culture), namely a decrease of *hrcA*, *ply* and *pspA* expression and an increase of *nanA*, *nanB*, *comA*, *comX* and *mgrA* (Fig. 1 and Table 1). The only exception being *nox*, which was less expressed on plastic than in all other conditions assayed (Table 1). For a subset of genes only, the expression values of biofilm bacteria were compared with stationary planktonic bacteria; gene expression patterns of pneumococci in the two different conditions were not identical, including an absence of upregulation of competence genes in planktonic stationary cells (data not shown).

### Biofilm formation is dependent on CSP

**Fig. 3:**
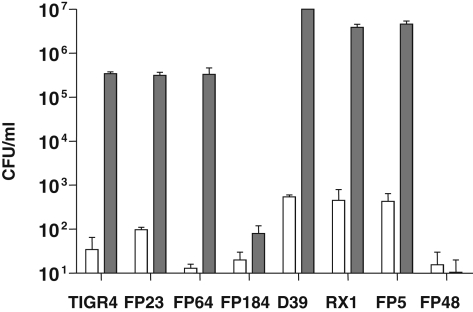
Viable counts of pneumococci attached to plastic. The pneumococcal strains TIGR4 and D39 and a series of isogenic mutants were evaluated for attachment to polystyrene microtiter wells. After 18 h of incubation in TSB the plates were washed and bacterial cells were detached by sonication. Counts of bacteria from wells with medium alone are shown by empty bars while counts of cells with CSP-supplemented medium are shown in grey (100 ng ml^−1^ of CSP2 for TIGR4 and 30 ng ml^−1^ of CSP1 for D39). Strains used in the experiment were the wild-type strains TIGR4 and D39, and their rough mutants FP23 and RX1, *comC* mutants FP64 and FP5, and *comD* mutants FP184 and FP48 respectively (Table 2).

The extent of biofilm formation was evaluated by determination of viable counts of pneumococci detached by sonication from wells of polystyrene plates after an overnight incubation. As mentioned above, data show that the number of cells attached to plastic increased 10 000 times over background when bacteria were incubated in CSP-supplemented medium (Fig. 3). Deletion of the capsule locus had no effect on biofilm formation, as viable counts of rough mutants showed identical ability to attach to plastic. To assay for the involvement of the competence regulon in biofilm formation, we compared TIGR4 and D39 to their isogenic mutants in the competence genes *comC* and *comD*(Table 2). Both the wild-type strains and their ComC-negative derivatives, FP64 and FP5, were able to form biofilms *in vitro* upon addition of exogenous CSP. Differently, the mutants for the CSP receptor ComD (FP184 and FP48) did not produce any biofilm structure (Fig. 3). Throughout these experiments, viable counts of attached cells of strain D39 and its mutants were higher than those of TIGR4, both in CSP-supplemented and CSP-free medium.

**Table 2:** Streptococcus pneumoniae strains.

Strain	Relevant properties	Reference
TIGR4	Type 4 genome strain, *comC2-comD2*	Tettelin *et al*. (2001)
D39	Type 2 Avery's strain, *comC1-comD1*	Pearce *et al*. (2002); Iannelli *et al*. (2004; 2005)
A66	Type 3 Avery's strain, *comC2-comD2*	Pearce *et al*. (2002); Iannelli *et al*. (2004; 2005)
G54	Type 19F genome strain, *comC1-comD1*	Pozzi *et al*. (1996); Dopazo *et al*. (2001); Iannelli *et al*. (2005)
ATCC6302	Type 2, *comC1-comD1*	Pozzi *et al*. (1996); Iannelli *et al*. (2005)
ATCC6303	Type 3, *comC1-comD4*	Pozzi *et al*. (1996); Iannelli *et al*. (2005)
ATCC6307	Type 7, *comC2-comD2*	Pozzi *et al*. (1996); Iannelli *et al*. (2005)
FP23	TIGR4 unencapsulated derivative; Km^R^	Oggioni *et al*. (2003); Pearce *et al*. (2002)
RX1	D39 unencapsulated derivative	Pearce *et al*. (2002)
FP5	Derivative of RX1, Δ*comC*; Cm^R^	Iannelli *et al*. (2005)
FP184	Derivative of TIGR4, *comD::aphIII*; Km^R^	Oggioni *et al*. (2004)
FP48	Derivative of RX1, *comD::aphIII*; Km^R^	This study
FP64	Derivative of TIGR4, Δ*comC*; Cm^R^	This study

**Fig. 4:**
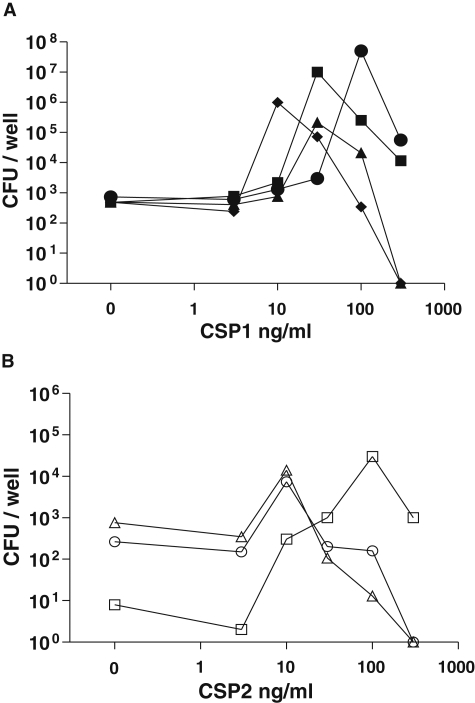
Concentration-dependent effect of CSP on attachment to plastic. The amount of biofilm formation of different *S. pneumoniae* strains incubated at increasing CSP concentrations (0, 3, 10, 30, 100 and 300 ng ml^−1^) was evaluated by determination of the viable counts of detached cells. A. Strains carrying the *comC*1 allele were incubated with CSP1; D39 (square), G54 (circle), ATCC6302 (triangle) and ATCC6305 (diamond). B. Strains carrying the *comC*2 allele were incubated with CSP2; TIGR4 (open square), A66 (open circle), and ATCC6307 (open triangle).

The optimal concentration of CSP that induced attachment to plastic was assayed by incubation of pneumococcal isolates of different serotype and CSP-pherotype with threefold serial dilutions of CSP, ranging from 3 to 300 ng ml^−1^ (Fig. 4). All strains were incubated using CSP1 or CSP2 according to the pherotypes of the strains (Pozzi *et al*., 1996). The total number of cells attached to the plastic varied form strain to strain and was found to be higher for strains carrying the *comC*1 allele (Fig. 4). Independently of the strain, CSP concentrations ranging from 10 to 100 ng ml^−1^ induced attachment, while levels of attached cells at 3 ng ml^−1^ and 300 ng ml^−1^ dropped to numbers detectable without addition of exogenous CSP. These data indicate that all strains tested have a narrow range in which exogenous CSP is optimal for bacterial attachment to plastic. The growth curve analysis by detecting turbidity of the liquid culture, overlaying the microtiter biofilms, was unchanged in the different conditions. The final turbidity in stationary phase was reduced for some strains at CSP concentrations that give maximal biofilm, but this was neither in direct relation to biofilm quantity nor a characteristic common to all strains (data not shown).

### The competence system has a role in pneumococcal virulence in mice

**Fig. 5:**
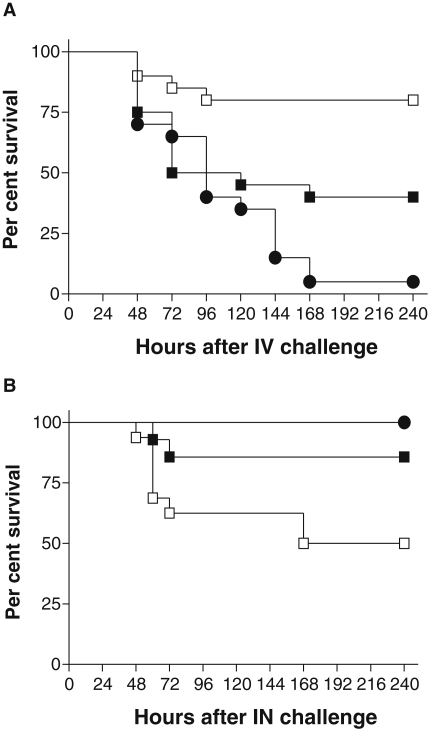
Impact of modulation of the competence system on pneumococcal virulence. The effect of inhibition and induction of the competence system on pneumococcal virulence was assayed in the murine infection models of sepsis and pneumonia. In both experiments three groups of mice were challenged with TIGR4 (control group, filled square), the *comD* mutant FP184 (competence negative strain, filled circle) and with TIGR4 treated with CSP2 (treatment group, open square). Results of the sepsis model (panel A) have been reported previously (Oggioni *et al*., 2004) and are reproduced with the permission of the publisher. In panel A mice were challenged IV and the treatment group (open square) received two IV doses of 1.3 μg CSP2 per mouse at time 0 and 24 h after challenge. Panel B reports IN challenge with a challenging dose of 2 × 10^5^ bacteria. In this pneumonia model the treatment group received 1.3 μg of CSP2 once together with the IN challenge. All experiments were performed in CD1 outbred mice (*n* = 20 IV; *n* = 2 × 16 IN) using the same frozen bacterial stocks for challenge. Differences in survival are statistically significant for the treatment groups both in the IV challenge in panel A (TIGR4 versus TIGR4 treated, *P = 0.012; FP184 versus TIGR4 treated, ***P < 0.0001; FP184 versus TIGR4, ns) and the IN challenge in panel B (TIGR4 treated versus FP184, **P = 0.0017; TIGR4 treated versus TIGR4, *P = 0.042; FP184 versus TIGR4, ns).

In order to assay if the divergent competence gene expression seen in different *in vitro* and *in vivo* environments was reflected in virulence, we did challenge experiments in the presence and absence of CSP. We chose IV and IN challenge as representatives of the two different patterns of competence gene expression (Fig. 1). The data from the IV infection model have already been published (Oggioni *et al*., 2004) but for clarity, are reported in Fig. 5A. In brief TIGR4 killed half of the mice, but administration of CSP, at challenge and after 24 h, increased survival of mice. Survival of mice challenged with the *comD* mutant was decreased compared with TIGR4 + CSP and TIGR4 alone. Repetition in this study of IV challenge with FP184, in presence and absence of CSP, confirmed the insensitivity to CSP and increased virulence of this mutant in the IV model (data not shown). Using transformation of pneumococci *in vitro* as detection tool to determine the stability of CSP *in vivo* we found that the half-life of CSP in blood was less than 1 min (data not shown).

Intranasal challenge was done with 2 × 10^5^ and with 2 × 10^6^ TIGR4 and FP184 drawn from the identical frozen stocks as for the previous experiment (Fig. 5). Groups of mice were challenged with the wild-type strain TIGR4 alone, TIGR4 mixed with CSP2, or with FP184. Each mouse was given a single dose of 1 μg CSP2 at challenge. At both infectious doses, FP184 was less virulent in this pneumonia model than TIGR4, as shown previously also by others (Bartilson *et al*., 2001; Hava and Camilli, 2002), and treatment with CSP increased virulence of TIGR4. The survival graph of the IN challenge with the lower infectious dose is shown in panel B of Fig. 5. Statistical analysis indicates that survival of mice challenged with TIGR4 (*P < 0.05) and challenged with FP184 (**P < 0.002) is significantly higher than survival of the group of mice challenged with TIGR4 and treated with CSP. When comparing the data of the pneumonia experiment with the data of the bacteraemia experiment it is obvious that induction of competence by CSP or inhibition of the competence cascade by mutation of *comD* has opposite effects in the two models. These virulence data show that in bacteraemic sepsis, where competence genes are found to be less expressed, further reduction of competence (i.e. in the *comD* mutant) results in more severe disease, while upregulation of competence (i.e. by using CSP) decreases the severity of disease. In contrast, when competence genes are more expressed, as in pneumonia and meningitis, reduction of competence results in decreased virulence, while upregulation of competence increases disease severity.

**Fig. 6:**
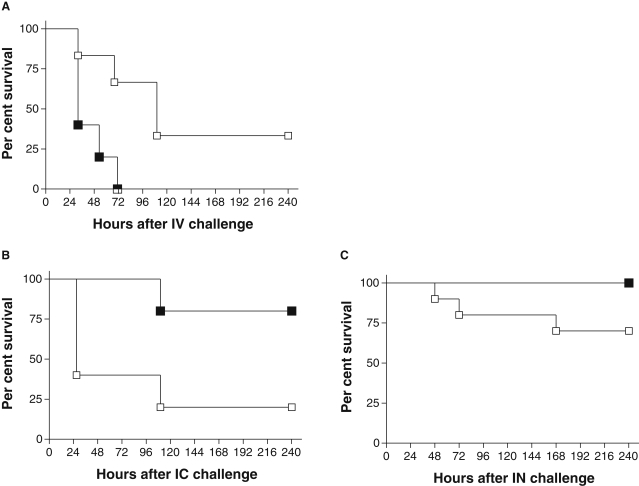
The effect of physical state of pneumococci on infectivity. A challenge experiment using in parallel sessile bacteria from a biofilm (open square) and planktonic cells from liquid culture (filled square) was done in the IV sepsis model, the IN pneumonia model and the IC meningitis model. In all experiments two groups of mice were challenged either with pneumococci from liquid culture (mid log phase) or from biofilm (24 h biofilm cells detached from microtiter plates). In panel A mice were challenged IV with 2 × 10^3^ cfu per mouse, panel B reports IC challenge with a dose of 2 × 10^2^ cfu and panel C reports IN challenge with a dose of 10^5^ cfu. All experiments were performed with strain TIGR4 in MF1 outbred mice (*n* = 6 sepsis; *n* = 5 meningitis; *n* = 10 pneumonia) using the same frozen pre-counted bacterial stocks for challenge. Differences in survival are statistically significant for the IV challenge in panel A (*P = 0.021) and for the IC challenge in panel B (*P = 0.045). The difference in virulence in panel C is not statistically significant (ns, *P* = 0.067).

In order to evaluate if the physical state *in vitro*, either planktonic or sessile, conferred characteristics with a direct impact on virulence we used bacteria from biofilm and form liquid culture for challenge. Pneumococci detached from microtiter wells and from mid log exponential growth were used in parallel for challenge in our IV sepsis model, the IN pneumonia model and the IC meningitis model. As shown in Fig. 6 bacteria from liquid culture were more virulent than bacteria form biofilm in the sepsis model, while to the contrary biofilm bacteria were more efficient in inducing meningitis and pneumonia. Differences in survival are statistically significant for the IV challenge (*P* = 0.021) and for the IC challenge (*P* = 0.045). In the IN experiment biofilm bacteria are more virulent than liquid culture bacteria, as in the IC model, but data are not statistically significant at this sample size (*P* = 0.067). It should be noted that none of the two challenge strains in this set of infections was mouse passaged, indicating that mouse passage is not essential for pneumococcal virulence.

## Discussion

The present work identifies two principal patterns of pneumococcal behaviour in the host during infection; one relative to bacteraemic sepsis and one to infection of tissues as lung and meninges. By gene expression profiling and phenotypic analysis the *in vivo* conditions of sepsis without primary focus of disease and tissue infection were correlated to the *in vitro* models of growth in liquid medium and to a newly developed biofilm model respectively. Recent data have suggested that the competence regulon could be involved in the regulation of such *in vivo* and *in vitro* systems (Oggioni *et al*., 2004). In this study, we examined the role of competence in the regulation of pneumococcal growth and virulence in both *in vitro* and *in vivo* environments by using gene expression data. The use of CSP and of CSP receptor knock-out strains enabled us to induce or inhibit virulence and growth phenotypes, and to demonstrate that the switch from planktonic life to a biofilm-like state is the key event in the pathogenesis of pneumococcal infection and that the quorum-sensing competence system is the prime regulatory mechanism in this event.

A crucial part of this work involved the set-up of a convenient biofilm model for *S. pneumoniae* in microtiter plates. This static biofilm model, described extensively for the related oral streptococci, is easier to perform than the more complex continuous culture biofilm models described so far for *S. pneumoniae* (Waite *et al*., 2001; Donlan *et al*., 2004; Allergucci *et al*., 2006). In the related species of oral streptococci, biofilm formation on polystyrene plates is described as being induced by CSP and inhibited in mutants for the CSP receptor ComD (Loo *et al*., 2000; Li *et al*., 2002; Cvitkovitch *et al*., 2003; Petersen *et al*., 2004; Qi *et al*., 2006). Our data show that only in the presence of exogenously added CSP, attached pneumococci could be detected on plastic surfaces and that recovery of such attached pneumococci was abolished when using *comD* mutants. The strict dependency on CSP distinguishes our pneumococcal microtiter biofilm model from the biofilm reactor model with continuous cultures, in which biofilm formation was independent from exogenously added CSP (Donlan *et al*., 2004). As pneumococci are aerotolerant anaerobes, biofilms are formed on the bottom of the wells, where they can be conveniently observed by light microscopy. Phase contrast microscopy showed irregular or ‘bleb-like’ structures independently of the serotype or pherotype of the strain used. The microscopic observation of blebs in phase contrast microscopy and the staining by alcian blue is compatible with formation of structures composed essentially by hydrated extracellular polysaccharide matrix material common to many biofilms (Donlan and Costerton, 2002). Upon fixation, microscopy reveals irregularly arranged bacterial cells, which are similar to microcolonies observed by others (Donlan and Costerton, 2002; Branda *et al*., 2005). The fact that biofilm is capsule independent, both in TIGR4 and D39, indicates that polymers other than the capsular polysaccharide are involved in structuring of pneumococcal biofilm (Donlan *et al*., 2004). The evidence of a role of the competence system in pneumococcal biofilm formation, as already found for oral streptococci (Loo *et al*., 2000; Li *et al*., 2002; Cvitkovitch *et al*., 2003; Petersen *et al*., 2004; Qi *et al*., 2006), allows to analyse its role in genetic transformation from an alternative point of view. During the natural process of competence for genetic transformation, cell density varies rapidly and is an important player in the quorum-sensing game. Differently in a bacterial biofilm, once build up, the cell density is not a main variable, leaving the control of its maintenance and destructuring to optimal signal concentration only. Under this aspect, it is not surprising why only a narrow range of CSP concentration is effective (Donlan and Costerton, 2002; Branda *et al*., 2005). Interestingly, the CSP concentrations effective in biofilm formation (30–100 ng ml^−1^ of CSP for cell densities of abut 5 × 10^8^ cfu ml^−1^) are similar in their signal to sensor ratio when compared with the concentrations inducing transformation (2–8 ng ml^−1^ of CSP for cell densities below 10^6^ cfu ml^−1^) (Havarstein *et al*., 1995). Recognizing this tight link between cell density and optimal CSP concentration, it is not surprising why natural competence is switched on only for few minutes in liquid culture during exponential growth.

The development of the biofilm model was performed with the overall aim to identify *in vitro* physiological states of the pneumococcus that mimic environments of *in vivo* infection (Beloin and Ghigo, 2005; Branda *et al*., 2005). To compare pneumococcal gene expression in *in vitro* conditions (liquid culture, submerged biofilm, agar plate) with *in vivo* models (pneumonia, meningitis and sepsis), we chose to use quantitative real time RT-PCR. This approach was chosen as this is the only technology able to quantify a range of targets ranging from 10^4^ (lung at 6 h after challenge) to over 10^8^ (severe sepsis), which is the range of concentrations bacteria reach in pneumococcal infection models (Kadioglu *et al*., 2000; 2002; Oggioni *et al*., 2004; Orihuela *et al*., 2004b; LeMessuier *et al*., 2006). Gene expression data were obtained for pneumococci collected both at end stage disease in models of pneumonia, meningitis and bacteraemic sepsis and during a time-course experiment of pneumonia. By monitoring pneumococcal gene expression in single organs of single mice, we identified two main gene expression patterns. In blood of mice inoculated intravenously (septic mice) pneumococci showed a pattern of gene expression that was very similar to that in mid exponential phase of growth in liquid culture. Three (*hrcA*, *ply* and *pspA*) out of the 29 genes monitored showed increased expression in this environment, when compared with pneumococci in tissue. Gene expression patterns in lung (IN challenge) or brain (IC challenge) were identical to each other but differed from that in blood (Table 1). In these tissue conditions surface enzymes involved in interaction with host components, namely the neuraminidases *nanA* and *nanB*, the three zinc metalloproteinases and enzymes related to oxidative stress and cell wall, were upregulated. The upregulation of *nanA* indicates a possible intriguing correlation of our biofilm phenotype to the transparent phase-variant pneumococci, which in turn are correlated to increased colonization capacity (King *et al*., 2004). A variety of regulators including *comE*, *comX*, *dtxR*, *mgrA* and *msmR* showed increased expression in this condition with respect to the liquid culture and sepsis. For nearly all the genes studied the expression pattern was identical in pneumococci recovered from tissue and from biofilm. This is remarkable when considering that a plethora of growth parameters were different in these situations. Some of the reported changes in gene expression possibly not be exclusive to biofilm or exponential growth, permitted us to obtain a recognizable pattern (Beloin and Ghigo, 2005; Cho and Caparon, 2005). This pattern provided a tool to characterize *in vitro* and *in vivo* models and to design a knowledge-based approach to study virulence *in vivo*. The fact that the challenge experiments confirm the predictions based on gene expression underline the solidity of the PCR data. The correlation between phenotypic modulation *in vitro* and *in vivo* and the identity in gene expression pattern indicate the biofilm model as a convenient *in vitro* tool to study pneumococci during tissue infection.

Our gene expression data fit quite well to those described by Orihuela for sepsis, while there is nearly no overlap with the evidences from the meningitis model (Orihuela *et al*., 2004b). This is most probably due to differences in host species, challenge dose and time of sampling in the two models. When comparing our data with those by LeMessuier, we found a good match between our tissue infection results and their data in the nose and partially in the lungs, but a poor matching with the data from blood (LeMessuier *et al*., 2006). Again, this might be due to differences in the models used (mouse strain, pneumococcal strain, route of infection). Due to the plethora of experimental conditions that influence infection models, extreme care must be taken in direct comparison of expression data of single genes *in vivo* in the absence of accompanying experimental evidence. This fact is underlined by a limited consensus of gene expression data reported so far on this subject (Marra *et al*., 2002; Ogunniyi *et al*., 2002; King *et al*., 2004; Orihuela *et al*., 2004b; LeMessuier *et al*., 2006).

Our previous work showing inhibition of pneumococcal sepsis in mice by CSP has gained a completely new perspective with the current data (Oggioni *et al*., 2004). The gene expression data demonstrate that bacteraemic sepsis (IV inoculum) is a disease in which pneumococci behave as they do in liquid culture in mid log phase of growth. Bacteraemic sepsis appears thus to be a disease in which bacteria tend to be in a planktonic state. The addition of CSP, which favours biofilm, shifts the pneumococcal population towards a suboptimal state and reduced fitness. In contrast, the shift of the whole population towards the planktonic state (*comD* mutant) renders the bacteria even more virulent in a disease were the planktonic state is the hallmark. The main challenge in this work was to evaluate if this *in vivo* situation, in which we could induce or reduce disease severity, could be repeated in a disease model with an opposite pattern of competence expression. We chose for this our IN infection model, as pneumococci in pneumonia were shown to upregulate competence genes. As predicted, the co-administration of CSP with bacteria significantly increased disease severity and morbidity in pneumonia. This is due to the fact that in a disease in which pneumococci behave as a biofilm, the forcing of this shift towards a biofilm state, by addition of synthetic CSP, exacerbates disease. In contrast, the *comD* mutant was less virulent in this model. These data are in accordance with data of others (Bartilson *et al*., 2001; Hava and Camilli, 2002), which show reduced virulence of the *comD* mutant in the TIGR4 background in pneumonia and in the D39 background in both pneumonia and in intraperitoneal disease. A decisive demonstration of the hypothesis that the condition of pneumococci in liquid culture and in biofilm was strongly related to their condition in sepsis and tissue infection, respectively, came from challenge experiments directly using bacteria in these diverse physical states. Planktonic bacteria were more efficient/fit at inducing sepsis, while pneumococci harvested from biofilms were more efficient/fit at inducing meningitis or pneumonia. These *in vivo* data demonstrate that there are at least two distinct physiologic states for pneumococci during infection of the host. The results also indicate that a shift of the bacterial population is always possible in a dynamic *in vivo* condition, in which population density and behaviour depends on inherently unstable quorums sensing signal molecules (Wang and Leadbetter, 2005). This datum, showing an upregulation of the competence system in tissue disease, provides also a new molecular justification to the historical data on genetic transformation of pneumococci during infection of the host (Griffith, 1928; Austrian, 1952; Conant and Sawyer, 1967).

The description of a biofilm state of pneumococci during tissue infection and of a planktonic state during bacteraemic sepsis is in accordance with the clinical and pathological description of these diseases (Kumar *et al*., 2004; Kasper *et al*., 2005). In the case of lobar pneumonia, which is the classical picture of pneumococcal pneumonia, disease develops over several days with a first phase of common respiratory disease, which is followed by rapid onset of symptoms and if untreated by solidification of alveolar space. During infection alveoli are filled with host cells, mucinous material and generally tend to collapse. In meningitis pathological findings are characterized mainly by inflammation of the pia and the arachnoid linings of the subarachnoidal space with shading of cells and bacteria into the cerebrospinal fluid (CSF). Both conditions are dependent on presence of bacteria at the site of damage and are characterized by marked local involvement of surface structures of body cavity linings. In contrast sepsis, in our case exemplified by a bacteraemia model without primary focus, is defined as a systemic inflammatory response syndrome (SIRS) with infectious cause, in which focal involvement of bacteria attached to surfaces is less relevant (Kasper *et al*., 2005). These clinical descriptions of pneumococcal disease fit very well to our observations, which assign distinct physiological states to the infecting bacterial population. Correlation of a biofilm state of infecting bacteria in acute pneumococcal infection is in accordance with the recent descriptions of biofilms formed by other bacterial pathogens responsible for acute disease as *Streptococcus pyogenes* (Cho and Caparon, 2005), *Haemophilus influenzae* (Ehrlich *et al*., 2006) and *Escherichia coli* (Anderson *et al*., 2006).

The fact that bacteria in nature are generally organized in biofilms is well recognized by experts working in environmental or industrial microbiology, but the importance of biofilms in medical microbiology is often underestimated. The best-studied medical biofilm examples include *Pseudomonas aeruginosa* pneumonia (controlled by the lasR/lasI system) (Storey *et al*., 1998), a variety of staphylococcal infections (controlled by the *agr* system) (Ji *et al*., 1995) and dental biofilms of oral streptococci (controlled by the *com* system). The quorum-sensing regulatory mechanisms of these models have been proposed as a novel drug target using derivatives of the quorum-sensing molecules as lead compounds (Mayville *et al*., 1999; Stephenson and Hoch, 2002). To this regard, it should be noted that our data indicate that regulatory systems may, in some occasions, be dangerous drug targets because as well as having a beneficial effect in one condition they can be detrimental in others.

In a three-step process consisting in (i) the description of gene expression patterns in models of infection, (ii) the identification of *in vitro* correlated models and (iii) the application of validated tools to modulate virulence *in vivo,* we have demonstrated that pneumococci during infection of the host are in different physical states depending on the disease. Pneumococci during infection of tissues (meningitis and pneumonia) resemble a biofilm-like mode of growth, while bacteria in the blood of septic mice resemble the *in vitro* model of growth as in liquid medium. The recognition of two diverse physiological conditions of pneumococci during infection and the description of *in vitro* correlated models may open new approaches for understanding the pathogenesis of pneumococcal infection.

## Experimental procedures

### Strains and growth conditions

Pneumococcal strains used in this work are reported in Table 2. Bacteria were grown in TSB (Becton Dickinson) or tryptic soy agar (TSA) supplemented with 3% horse blood at 37°C in a CO_2_-enriched atmosphere. Bacterial stocks were prepared from mid log cultures and stored frozen at −80°C in 10% glycerol. Antibiotics where appropriate were used at the following concentrations: kanamycin 500 μg ml^−1^, spectinomycin 100 μg ml^−1^, chloramphenicol 3 μg ml^−1^ and novobiocin 10 μg ml^−1^.

### Mutant construction

Isogenic mutants were constructed by gene SOEing as already described (Pearce *et al*., 2002; Iannelli and Pozzi, 2004). Deletion of *comC* was obtained by transforming TIGR4 with a genetic cassette amplified from FP5 (Iannelli *et al*., 2005), and the resulting strain was denominated FP64. In FP48, insertion of a kanamycin-resistant mariner minitransposon in *comD* was obtained by transforming RX1 as already reported (Oggioni *et al*., 2004). Mutants were selected by multilayer plating as previously described (Iannelli and Pozzi, 2004).

### Biofilm model

Bacteria were grown in flat-bottom polystyrene tissue culture plates (96-well plates; Sarstedt, USA). Frozen pneumococcal cultures (about 1 × 10^8^ cfu ml^−1^) were diluted 1:100 in 200 μl of TSB with or without the addition of CSP (Inbios S.r.l. Napoli, Italy) at various concentrations (0–300 ng ml^−1^). Plates were incubated for 18–24 h at 37°C in a CO_2_-enriched atmosphere. Turbidity of bacterial cultures (OD_590_) was measured by using the VERSAmax Microplate Reader ELISA apparatus (Molecular Devices, USA). To remove planktonic bacteria, wells were washed four times with ice-cold TSB, and added with 100 μl of TSB containing 10% glycerol. To detach biofilm bacteria, plates were subjected to a 2 s sonication in a water bath (Transonic 460, Elma, Germany). Detached cells (100 μl) were recovered from plates for cfu counts and real time RT-PCR or directly frozen. Sonication for 5 or 10 s did not increase cell recovery, while sonication for 30 s killed bacteria.

### Light microscopy of biofilm

Pneumococcal biofilm for microscopic examination was grown in 6 well flat-bottom polystyrene tissue culture plates (Sarstedt, USA) in 2 ml of CSP-supplemented TSB for 18 h in anaerobiosis. After washing the plates with TSB, wells were air-dried, fixed with methanol for 1 min, stained with 2% alcian blue 8GX (Sigma) for 10 min, rinsed, stained with 4% crystal violet (Sigma) for 5 s, rinsed again, and finally dried. Phase contrast microscopy was carried out on washed samples without fixation and staining. Examination of biofilm morphology was done by using a Leica DM1000 Microscope (Leica Microsystems Wetzlar, Germany; 40× or 100× magnification) and a Leica DFC digital camera (Leica DFC, Cambridge, UK).

### Models of infection

Meningitis, pneumonia and sepsis models of infection were used. MF1 and CD1 mice were purchased by Harlan Nossan (Correzzana, Italy and Bichester, UK) and Charles River Italia (Calco, Italy) respectively. Experiments were carried out either at the University of Siena or at the University of Leicester according to the respective national and institutional guidelines. Four different types of experiments were performed: (i) end-stage disease by using meningitis, pneumonia and sepsis models to analyse gene expression *in vivo* (MF1 mice; University of Siena), (ii) time-course pneumonia model for gene expression analysis *in vivo* (MF1 mice; University of Leicester), (iii) pneumonia model to study the effect(s) of CSP *in vivo* (CD1 mice; University of Siena) and (iv) an assay for infectivity of pneumococci harvested from either biofilm or from liquid culture in models of pneumonia, meningitis and sepsis (MF1 mice; University of Siena). Mouse-passaged pneumococci, prepared as previously described (Kadioglu *et al*., 2000), were used for inocula. Prior to infection, bacteria were thawed at room temperature, harvested by centrifugation and resuspended in sterile phosphate-buffered saline, pH = 7.4 (PBS). After infection, mice were regularly monitored for clinical signs (starry fur, hunched appearance and lethargy) throughout the experiment. Mice before becoming severely lethargic during the course of the experiment were humanely sacrificed. In experiment (i), mice were infected with TIGR4 either via the intracranial–subarachnoidal (IC) route with 10^5^ cfu (total volume of 30 μl; *n* = 5) (Chiavolini *et al*., 2004), or the IN route with 5 × 10^7^ cfu (15 μl per nostril; *n* = 5) (Chiavolini *et al*., 2003), or the IV route with 4 × 10^7^ cfu (200 μl; *n* = 5) (Iannelli *et al*., 2004). All mice becoming symptomatic after IC or IN inoculum were confirmed to be bacteraemic. Mice with meningitis were sacrificed between 18 and 32 h from infection, animals with pneumonia between 39 and 57 h, while septicaemic mice were killed between 40 and 57 h. Brains, lung lavages and blood samples were collected. In experiment (ii), mice (*n* = 12) were lightly anaesthetized with 2.5% (v/v) fluo-thane (AstraZeneca, Macclesfield, UK) over oxygen (1.5–2 l min^−1^) prior to IN infection with 50 μl of PBS containing 1 × 10^6^ cfu of TIGR4. At set time-points, groups of mice were deeply anaesthetized with 5% (v/v) fluothane. Blood, bronchoalveolar lavages and lung samples were collected. In experiment (iii), CD1 mice were infected IN as described (Oggioni *et al*., 2004). Three groups of mice (*n* = 16/group) were challenged with either TIGR4, or FP184, or with TIGR4 and CSP2 (1.3 μg mouse^−1^). Experiments were performed with two different bacterial doses (2 × 10^5^ and 2 × 10^6^ cfu). In experiment (iv), the inoculum preparation differed from the previous three ones. Bacteria were harvested either from mid-log phase cultures by centrifugation or from the bottom of microtiter plates by sonication. Both types of inocula were resuspended in TSB glycerol, stored frozen as aliquots, and diluted in TSB for challenge. Pneumococci were not mouse-passaged prior to infection. MF1 mice were infected either IC with 10^2^ cfu (*n* = 5), or IN with 2 × 10^5^ cfu (*n* = 10), or IV with 10^3^ cfu (*n* = 6). All mice that became severely symptomatic were humanely sacrificed. Statistical analysis of survival was done by using the chi-square test.

### Sample collection for RT-PCR

Bacteria were sampled from liquid broth, solid medium and infected mice. Pneumococcal cells grown in TSB until mid log phase (OD_590_ = 0.2) were chilled on ice, harvested by centrifugation, and the bacterial pellet was stored frozen at −80°C until use. Bacteria cultured on TSA plates were collected after 18 h of incubation at 37°C. Plates were incubated in a candle jar with a burning candle to avoid excessive oxidative stress or pH drop resulting from adding CO_2_ for anaerobic incubation. Bacteria were recovered from agar in 1 ml of ice-cold TSB, put on ice, collected by centrifugation and frozen at −80°C. Pneumococci were detached from biofilms as described above. Mouse samples were obtained from individual animals immediately after the sacrifice. Brains homogenates, lung lavages and blood were collected from mice with meningitis, pneumonia and sepsis respectively. As the amount of CSF obtainable from mice does not exceed few microlitres, we chose to use whole brain homogenates as representative samples for evaluation of gene expression in meningeal infections. Blood was obtained by retrorbital bleeding into tubes containing 1/10 volume of 0.1 M sodium citrate; brains were homogenated in 1 ml of ice-cold PBS, and lung washes were done with 1 ml of PBS. All samples were put on ice and subjected immediately to a low speed centrifugation (800 *g*, 5 min, 4°C). The supernatant, containing the bacteria, was transferred into a new tube and centrifuged (10 000 *g*, 5 min, 4°C). The pellet of all samples was resuspended in 100 μl of TE buffer (Tris 10 mM, EDTA 1 mM, pH 7.5) before storage at −80°C.

### RNA extraction, retrotranscription and quantitative real time RT-PCR

RNA was extracted by using ‘SV Total RNA Isolation System Kit’ (Promega) according to the manufacturer's instructions with few modifications: treatment with DNAse was shortened to 10 min and elution of RNA was in 50 μl of nuclease-free H_2_O. Samples were stored frozen at −80°C. Retrotranscription was carried out by using the ‘ImProm-II Reverse Transcriptase Kit’ (Promega) according to the manufacturer's instructions as described. Briefly, annealing was performed at 25°C for 10 min and extension at 37°C for 1 h. Samples were inactivated at 70°C for 15 min and immediately subjected to real time PCR. Quantitative real time PCR was performed as previously described (Oggioni *et al*., 2004) in a Light Cycler apparatus (Roche) by using the ‘Light Cycler DNA-Master SYBR Green I Kit’ (Roche). As PCR template, 2 μl of cDNA was used. Primer efficiency was verified by using serial dilution of cDNA ranging from 10^2^ to 10^6^ target copies per reaction (10^4^−10^8^ target copies per sample), and only oligonucleotides with comparable efficiency were chosen. Primers spanning 100–150 bp segments were designed by standard procedures and are reported as *Supplementary material* (Table S1). Primers for *gyrB* (SP0806), *comA* (SP0042), *lytA* (SP1937), *comX1* (SP0014) and *dprA* (SP1266) were as previously described (Oggioni *et al*., 2004). The relative gene expression was analysed by using the 2^–ΔΔCT^ method (Livak and Schmittgen, 2001). The reference gene was *gyrB* and the reference condition was exponential phase of growth in TSB. Gene expression in TSA plates and *in vivo* is represented as the increment/decrement (folds) towards expression in TSB. For each analysis, three to five distinct biological replicas were done, and quantitative data were expressed as mean ± SD. Values of fold change in gene expression above 2 or below 0.5 as determined by the 2^–ΔΔCT^ method were considered significant.

## Supplementary material

The following supplementary material is available for this article online:

Table S1. Primer pairs used for real time PCR of *S. pneumoniae* TIGR4.

This material is available as part of the online article from http://www.blackwell-synergy.com

## References

[b1] Afessa B, Greaves WL, Frederick WR (1995). Pneumococcal bacteremia in adults: a 14-year experience in an inner-city hospital. Clin Infect Dis.

[b2] Allergucci M, Hu FZ, Shen K, Hayes J, Ehrlich GD, Post JC, Sauer K (2006). Phenotypic characterization of *Streptococcus pneumoniae* biofilm development. J Bacteriol.

[b3] Alloway JL (1932). The transformation *in vitro* of R pneumococci into S forms of different specific types by the use of filtered pneumococcus extracts. J Exp Med.

[b4] Anderson GG, Dodson KW, Hooton TM, Hultgren SJ (2006). Intracellular bacterial communities of uropathogenic *Escherichia coli* in urinary tract pathogenesis. Trends Microbiol.

[b5] Aszkenasy OM, George RC, Begg NT (1995). Pneumococcal bacteraemia and meningitis in England and Wales 1982–92. Commun Dis Rep.

[b6] Austrian R (1952). Observations on the transformation of pneumococcus *in vivo*. Bull John Hopkins Hosp.

[b7] Bartilson M, Marra A, Christine J, Asundi JS, Schneider WP, Hromckyj AE (2001). Differential fluorescence induction reveals *Streptococcus pneumoniae* loci regulated by competence stimulatory peptide. Mol Microbiol.

[b8] Beloin C, Ghigo JM (2005). Finding gene-expression patterns in bacterial biofilms. Trends Microbiol.

[b9] Blue CE, Paterson GK, Kerr A, Berge M, Claverys JP, Mitchell TJ (2003). ZmpB, a novel virulence factor of *Streptococcus pneumoniae* that induces tumor necrosis factor alpha production in the respiratory tract. Infect Immun.

[b10] Bornstein DL, Schifferman G, Bernheimer HP, Austrian R (1968). Capsulation of pneumococcus with soluble C-like (Cs) polysaccharide. I. Biological and genetic properties of Cs pneumococcal strains. J Exp Med.

[b11] Branda SS, Vik S, Friedman L, Kolter R (2005). Biofilms: the matrix revised. Trends Microbiol.

[b12] Chiavolini D, Memmi G, Maggi T, Iannelli F, Pozzi G, Oggioni MR (2003). The three extra-cellular zinc metalloproteinases of *Streptococcus pneumoniae* have a different impact on virulence in mice. BMC Microbiol.

[b13] Chiavolini D, Tripodi S, Parigi R, Oggioni MR, Blasi E, Cintorino M (2004). Method for inducing experimental pneumococcal meningitis in outbred mice. BMC Microbiol.

[b14] Cho KH, Caparon MG (2005). Patterns of virulence gene expression differ between biofilm and tissue communities of *Streptococcus pyogenes*. Mol Microbiol.

[b15] Conant JE, Sawyer WD (1967). Transformation during mixed pneumococcal infection of mice. J Bacteriol.

[b16] Cvitkovitch DG, Li YH, Ellen RP (2003). Quorum sensing and biofilm formation in streptococcal infections. J Clin Invest.

[b17] Dagkessamanskaia A, Moscoso M, Henard V, Guiral S, Overweg K, Reuter M (2004). Interconnection of competence, stress and CiaR regulons in *Streptococcus pneumoniae*: competence triggers stationary phase autolysis of *ciaR* mutant cells. Mol Microbiol.

[b18] Donlan RM, Costerton JW (2002). Biofilms: survival mechanism of clinically relevant microorganism. Clin Microbiol Rev.

[b19] Donlan RM, Piede JA, Heyes CD, Sanii L, Murga R, Edmonds P (2004). Model system for growing and quantifying *Streptococcus pneumoniae* biofilms *in situ* and in real time. Appl Environ Microbiol.

[b20] Dopazo J, Mendoza A, Herrero J, Caldara F, Humbert Y, Friedli L (2001). Annotated draft genomic sequence from *Streptococcus pneumoniae* type 19F clinical isolate. Microb Drug Resist.

[b21] Echenique JR, Trombe MC (2001). Competence repression under oxygen limitation through the two component MicAB signal transducing system in *Streptococcus pneumoniae* and involvement of the PAS domain of MicB. J Bacteriol.

[b22] Ehrlich GD, Veeh R, Wang X, Costerton JW, Hayes JD, Hu FZ (2006). Mucosal biofilm formation on middle-ear mucosa in the chinchilla model of otitis media. JAMA.

[b23] Griffith F (1928). The significance of pneumococcal types. J Hyg.

[b24] Hava DL, Camilli A (2002). Large-scale identification of serotype 4 *Streptococcus pneumoniae* virulence factors. Mol Microbiol.

[b25] Hava DL, LeMieux J, Camilli A (2003). From the nose to the lung: the regulation behind *Streptococcus pneumoniae* virulence factors. Mol Microbiol.

[b26] Havarstein LS, Coomaraswamy G, Morrison DA (1995). An unmodified pheromone induces competence for genetic transformation in *Streptococcus pneumoniae*. Proc Natl Acad Sci USA.

[b27] Iannelli F, Pozzi G (2004). Method for introducing specific and unmarked mutations into the chromosome of *Streptococcus pneumoniae*. Mol Biotechnol.

[b28] Iannelli F, Chiavolini D, Ricci S, Oggioni MR, Pozzi G (2004). Pneumococcal surface protein C (PspC) contributes to sepsis caused by *Streptococcus pneumoniae*. Infect Immun.

[b29] Iannelli F, Oggioni MR, Pozzi G (2005). Sensor domain of histidine kinase ComD confers competence pherotype specificity in *Streptococcus pneumoniae*. FEMS Microbiol Lett.

[b30] Ji G, Beavis RC, Novick RP (1995). Cell density control of staphylococcal virulence mediated by an octapeptide pheromone. Proc Natl Acad Sci USA.

[b31] Kadioglu A, Gingles NA, Grattan K, Kerr A, Mitchell TJ, Andrew PW (2000). Host cellular immune response to pneumococcal lung infection in mice. Infect Immun.

[b32] Kadioglu A, Taylor S, Iannelli F, Pozzi G, Mitchell TJ, Andrew PW (2002). Upper and lower respiratory tract infection by *Streptococcus pneumoniae* is affected by deficiency of pneumolysin and by differences in serotype. Infect Immun.

[b33] Karstaedt AS, Khoosal M, Crewe-Brown HH (2001). Pneumococcal bacteremia in adults in Soweto, South Africa, during the course of a decade. Clin Infect Dis.

[b34] Kasper DL, Braunwald E, Fauci AS, Hauser SL, Longo DL, Jameson JL (2005). Harrison's Principles of Internal Medicine.

[b35] King SJ, Hippe KR, Gould JM, Bae D, Peterson F, Cline RT (2004). Phase variable desialylation of host proteins that bind to *Streptococcus pneumoniae in vivo and* protect the airway. Mol Microbiol.

[b36] Kumar V, Abbas AK, Fausto N (2004). Robbins and Contran Pathologic Basis of Disease 7E.

[b37] Lau GW, Haataja S, Lonetto M, Kensit SE, Marra A, Bryant AP (2001). A functional genomic analysis of type 3 *Streptococcus pneumoniae* virulence. Mol Microbiol.

[b38] LeMessuier KS, Ogunniyi DA, Paton JC (2006). Differential expression of key pneumococcal virulence genes *in vivo*. Microbiology.

[b39] Li YH, Tang N, Aspiras MB, Lau PCY, Lee JH, Ellen RP, Cvitkovitch DG (2002). A quorum-sensing signaling system essential for genetic competence in *Streptococcus mutans* is involved in biofilm formation. J Bacteriol.

[b40] Livak KJ, Schmittgen TD (2001). Analysis of relative gene expression data using real-time quantitative PCR and the 2 (-Delta Delta C (T)) Method. Methods.

[b41] Loo CY, Corliss DA, Ganeshkumar N (2000). *Streptococcus gordonii* biofilm formation: identification of genes that code for biofilm phenotypes. J Bacteriol.

[b42] Manco S, Hernon F, Yesilkaya H, Paton JC, Andrew PW, Kadioglu A (2006). Pneumococcal neuraminidases A and B both have essential roles to play during infection of the respiratory tract and sepsis. Infect Immun.

[b43] Marra A, Asundi J, Bartilson M, Lawson S, Fang F, Christine J (2002). Differential fluorescence induction analysis of *Streptococcus pneumoniae* identifies genes involved in pathogenesis. Infect Immun.

[b44] Mascher T, Zahner D, Merai M, Balmelle N, de Saizieu A, Hakenbeck R (2003). The *Streptococcus pneumoniae cia* regulon: ciaR target sites and transcription profile analysis. J Bacteriol.

[b45] Mayville P, Guangyong JI, Beavis R, Yang H, Goger M, Novick RP, Muir TW (1999). Structure-activity analysis of synthetic autoinducing thiolactone peptides from *Staphylococcus aureus* responsible for virulence. Biochemistry.

[b46] Oggioni MR, Memmi G, Maggi T, Chiavolini D, Iannelli F, Pozzi G (2003). Pneumococcal zinc metalloproteinase ZmpC cleaves human matrix metalloproteinase 9 and is a virulence factor in experimental pneumonia. Mol Microbiol.

[b47] Oggioni MR, Iannelli F, Ricci S, Chiavolini D, Parigi R, Trappetti C (2004). Antibacterial activity of a competence-stimulating peptide in experimental sepsis caused by *Streptococcus pneumoniae*. Antimicrob Agents Chemother.

[b48] Ogunniyi DA, Giammarinaro P, Paton JC (2002). The genes encoding the virulence-associated proteins and the capsule of *Streptococcus pneumoniae* are upregulated and differentially expressed in vivo. Microbiology.

[b49] Orihuela CJ, Gao G, Francis KP, Yu J, Tuomanen E (2004a). Tissue-specific contributions of pneumococcal virulence factors to pathogenesis. J Infectious Dis.

[b50] Orihuela CJ, Radin JN, Sublett JE, Gao G, Kaushal D, Tuomanen E (2004b). Microarray analysis of pneumococcal gene expression during invasive disease. Infect Immun.

[b51] Pearce BJ, Iannelli F, Pozzi G (2002). Construction of new unencapsulated (rough) strains of *Streptococcus pneumoniae*. Res Microbiol.

[b52] Petersen FC, Pecharki D, Scheie AA (2004). Biofilm mode of growth of *Streptococcus intermedius* favored by a competence-stimulating signaling peptide. J Bacteriol.

[b53] Peterson SN, Sung CK, Cline R, Desai BV, Snesrud EC, Luo P (2004). Identification of competence pheromone responsive genes in *Streptococcus pneumoniae* by use of DNA microarrays. Mol Microbiol.

[b54] Polissi A, Pontiggia A, Feger G, Altieri M, Mottl H, Ferrari L, Simon D (1998). Large-scale identification of virulence genes from *Streptococcus pneumoniae*. Infect Immun.

[b55] Pozzi G, Masala L, Iannelli F, Manganelli R, Havarstein LS, Piccoli L (1996). Competence for genetic transformation in encapsulated strains of *Streptococcus pneumoniae*: two allelic variants of the peptide pheromone. J Bacteriol.

[b56] Qi F, Kreth J, Levesque CM, Kay O, Mair RW, Shi W (2006). Peptide pheromone induced cell death of *Streptococcus mutans*. FEMS Microbiol Lett.

[b57] Stephenson K, Hoch JA (2002). Two-component and phosphorelay signal-transduction systems as therapeutic targets. Curr Opin Pharmacol.

[b58] Storey DG, Ujack EE, Rabin HR, Mitchell I (1998). *Pseudomonas aeruginosa lasR* transcription correlates with the transcription of *lasA*, *lasB*, and *toxA* in chronic lung infections associated with cystic fibrosis. Infect Immun.

[b59] Tettelin H, Nelson KE, Paulsen IT, Eisen JA, Read TD, Peterson S (2001). Complete genome sequence of a virulent isolate of *Streptococcus pneumoniae*. Science.

[b60] Tomasz A (1965). Control of the competent state in *Pneumococcus* by a hormone-like cell product: an example for a new type of regulatory mechanism in bacteria. Nature.

[b61] Tomasz A, Hotchkiss RD (1964). Regulation of the transformability of pneumococcal cultures by macromolecular cell products. Proc Natl Acad Sci USA.

[b62] Waite RD, Struthers JK, Dowson CG (2001). Spontaneous sequence duplication within an open reading frame of the pneumococcal type 3 capsule locus causes high-frequency phase variation. Mol Microbiol.

[b63] Wang YJ, Leadbetter JR (2005). Rapid acyl-homoserine lactone quorum signal biodegradation in diverse soils. Appl Environ Microbiol.

