# Supplementary material for: Switch from planktonic to sessile life: a major event in pneumococcal pathogenesis
Source: Mol Microbiol. 2006 Aug 1;61(5):1196–210. doi: 10.1111/j.1365-2958.2006.05310.x (PMC1618759; doi:10.1111/j.1365-2958.2006.05310.x)
Supplement: Table S1. — Primer pairs used for real time PCR of S. pneumoniae TIGR4. [file mmi0061-1196-TableS1.doc]

Supplementary materials: Primer pairs used for real time PCR of *S. pneumoniae* TIGR4

| Primer | Sequence | Target gene  (TIGR4 annotation) | Lenght of amplicon | | |
| --- | --- | --- | --- | --- | --- |
| GM210 | GATGAGTCAAGGGATTCAATCG | *zmpC* (SP0071) | 118 bp | | |
| CT21 | AAGGGCCTCTACCAGCAAG |  | |  |  |
| CT13 | GTAGCACTAGCGAAGAAAGA | *pspA* (SP0117) | 107bp | | |
| CT14 | ACTTGATGTTGAGCAGTAGCA |  | |  |  |
| CT58 | GTCACGCCAGTCTAACCATT | *regR* (SP0330) | 118 bp | | |
| CT59 | GCCCAACAGTTAGGGATAA |  | |  |  |
| CT 108 | TCCAGAGGTTCCCAGCAAG | pbp2x (SP0336) | 114 bp | | |
| CT 109 | ACAACCAGTCCCAATCGTAG |  | |  |  |
| CT64 | TATGTTCGCTTGATTGGG | *luxS* (SP0340) | 105 bp | | |
| CT65 | GCCGGCAGTAGGGATAGAGT |  | |  |  |
| CT32 | GCGGTATTTACCCTAGAC | *hrcA* (SP0515) | 150 bp | | |
| CT33 | CCGAATCTTGTAGTGAATATCT |  | |  |  |
| CT54 | TTACAGCGAGTTTAGAGGAGGT | *blpR* (SP0526) | 150 bp | | |
| CT55 | GGGCGATTAGACAACTTC |  | |  |  |
| CT44 | ATCCCTCATGCGTTCAATTATT | *prtA* (SP0641) | 158 bp | | |
| CT45 | CCATCTATGCCGTTAAAGT |  | |  |  |
| CT38 | AATGAGCGCTAGAAATGTTGT | *zmpB* (SP0664) | 124 bp | | |
| CT49 | ATTAAATAATGGATGTTCCAAT |  | |  |  |
| CT74 | TGAAGACCTTGAAGCCTT | *sodA* (SP0766) | 084 bp | | |
| CT75 | TGTCCGCCACCATTGTTGAT |  | |  |  |
| CT46 | GATGTTATGCAGGTATTTGATG | *ciaR* (SP0798) | 157 bp | | |
| CT47 | TAATCAGAACTGGTGTCGTAAT |  | |  |  |
| CT42 | TGGCAGATTCAGAGCTATCATC | *iga* (SP1154) | 159 bp | | |
| CT43 | GCCTCTCATTCTTGCTTCC |  | |  |  |
| CT60 | TGCTAAGACCATTCGTAAGACA | *micA* (SP1227) | 162 bp | | |
| CT61 | CGCAGAAGAGCTTTAACACGC |  | |  |  |
| CT76 | ATTGACGGTGCTGAAGGCTTG | *nox* (SP1469) | 120 bp | | |
| CT77 | CGCTGTAACTACTTTGTTA |  | |  |  |
| CT78 | AGGAAATTGCGGCTCGCAT | *dtxR smrB* (SP1638) | 149 bp | | |
| CT79 | ACGATAGAGCTCAGAGACCAG |  | |  |  |
| CT 106 | TAACTACGATGTCAAGAGCA | ddlA (SP1671) | 135 bp | | |
| CT 107 | ACAGCCACCACATCATCACT |  | |  |  |
| CT66 | AAGCTTTGTTTGGCCTATCT | *nanB* (SP1687) | 107 bp | | |
| CT67 | TTGGGCGTCTCTTATTTC |  | |  |  |
| CT72 | AGCAACCTCTGGCAAATGAA | *nanA* (SP1693) | 158 bp | | |
| CT73 | ATAGTAATCTCTTGGAATT |  | |  |  |
| CT56 | TATCGTTCGGATAACAGAT | *stkP* (SP1732) | 152 bp | | |
| CT57 | CCAAGAGAATTTGTCCCAT |  | |  |  |
| CT62 | CGCATTATTAATACCGATG | *mgrA* (SP1800) | 161 bp | | |
| CT63 | CGATAGAGCGAAGATGAAC |  | |  |  |
| CT80 | GGTCCAGCCATTCGTGATACAT | *msmR araC* (SP1899) | 187 bp | | |
| CT81 | CCAGTGATTCCTAACCAG |  | |  |  |
| CT17 | GCAAATAAAGCAGTAAAT | *ply* (SP1923) | 215 bp | | |
| CT18 | CCAGGATAGAGGCGAC |  | |  |  |
| CT26 | GCTGATGACTACCTGACCAAG | *pnpR* (SP2082) | 150 bp | | |
| CT27 | GTATACTTTCAAGGTCCCAAAT |  | |  |  |
| IF131 | GAAAAGTACATTATTCAATTCGTAA | *pspC* (SP2190) | 086 bp | | |
| IF132 | ATGAACCACACTTCCCATAA |  | |  |  |
| CT68 | CGGAGGCTACATTCGTATA | *ctsR* (SP2195) | 099 bp | | |
| CT69 | ATTTCTTGACTGACTCGCTCA |  | |  |  |
| CT19 | TAAATCAGCTTTATTTCCTAG | *comE* (SP2235) | 232 bp | | |
| CT20 | GTGTAGAAGATATTTTGC |  | |  |  |
